# Epidemiology of metabolic syndrome

**DOI:** 10.1007/s00424-024-03051-7

**Published:** 2025-01-25

**Authors:** Iris Pigeot, Wolfgang Ahrens

**Affiliations:** 1 Leibniz Institute for Prevention Research and Epidemiology – BIPS, Bremen, Germany; 2 Institute of Statistics, Faculty of Mathematics and Computer Science, University of Bremen, Bremen, Germany

**Keywords:** Cardio-metabolic risk factors, Dyslipidemia, Hypertension, Insulin resistance, Obesity, Prevalence

## Abstract

The global increase of overweight and obesity in children and adults is one of the most prominent public health threats, often accompanied by insulin resistance, hypertension, and dyslipidemia. The simultaneous occurrence of these health problems is referred to as metabolic syndrome. Various criteria have been proposed to define this syndrome, but no general consensus on the specific markers and the respective cut-offs has been achieved yet. As a consequence, it is difficult to assess regional variations and temporal trends and to obtain a comprehensive picture of the global burden of this major health threat. This limitation is most striking in childhood and adolescence, when metabolic parameters change with developmental stage. Obesity and related metabolic disorders develop early in life and then track into adulthood, i.e., the metabolic syndrome seems to originate in the early life course. Thus, it would be important to monitor the trajectories of cardio-metabolic parameters from early on. We will summarize selected key studies to provide a narrative overview of the global epidemiology of the metabolic syndrome while considering the limitations that hinder us to provide a comprehensive full picture of the problem. A particular focus will be given to the situation in children and adolescents and the risk factors impacting on their cardio-metabolic health. This summary will be complemented by key findings of a pan-European children cohort and first results of a large German adult cohort.

## Introduction

Starting in high income countries and driven by unfavorable health behaviors like physical inactivity and unhealthy diets the increasing prevalence of metabolic disorders has reached pandemic dimensions [43]. The global increase of overweight and obesity in children and adults is probably the most obvious health threat which is often accompanied by insulin resistance, hypertension, and dyslipidemia. All of these disorders increase the risk for cardiovascular diseases and thus contribute substantially to the global burden of non-communicable diseases. Since these cardiovascular risk factors often cluster in one individual, their joint occurrence is characteristic of an overriding syndrome and has thus been called metabolic syndrome (MetS) [4]. However, numerous diverse criteria regarding the markers to be measured and their cut-offs are currently in use to define the MetS, which makes it difficult to assess regional variations and temporal trends and to obtain a picture of the global burden of this major health threat [36]. Since obesity and related metabolic disorders develop early in life and then track into adulthood, it would be important to monitor their occurrence from early on. This calls for an adequate definition of MetS in children that considers the changes in body size and body composition during growth and developmental transitions during puberty. Such a definition that considers the growth-dependent age- and sex-specific changes of cardio-metabolic markers has been proposed by Ahrens et al. [1] for pre-pubertal children, but corresponding reference values are still missing for adolescents [13]. For this reason, many authors were left with adult criteria to describe the population-burden of MetS in children and adolescents, rendering their results questionable.

With this paper, we will summarize the results of selected key studies to provide a narrative overview of the global epidemiology of the MetS while considering the limitations that hinder us to provide a comprehensive full picture of the problem. A particular focus will be given to the situation in children and adolescents and the risk factors impacting on their cardio-metabolic health as it seems that the MetS originates in the early life course. This summary will be complemented by key findings of a pan-European children cohort and first results of a large German adult cohort.

## Metabolic disorders during childhood

### Worldwide prevalence of the metabolic syndrome in children and adolescents

Without claiming completeness, this section will briefly summarize some studies on the prevalence of the MetS in childhood and adolescence. As outlined in the introduction, such a summary is hampered by the fact that (1) in most pediatric studies, adult criteria or their adaptations were used to define the MetS. This makes it difficult to quantify the proportion of children with MetS and to assess its clinical impact [44]. Further challenges are (2) that diverse definitions were in use as already problematized in a review by Ford and Li [19] where 40 different definitions of the MetS were identified in 27 publications; and (3) that most studies only consider children and adolescents above the age of ten, a fact that may be due to ethical concerns of drawing blood from very young children who are healthy. For a detailed comparison of the various criteria used to define the MetS in children and adolescents we refer to [44]. However, data which are comparable are urgently needed to learn more about the worldwide situation and the temporal trends of the prevalence of MetS in children and adolescents.

It is well known that the prevalence of childhood obesity and overweight has increased worldwide. According to a pooled analysis of 2416 population-based studies with measurements of height and weight including 31.5 million children and adolescents aged 5–19 years [32], the rising trends in the prevalence of overweight and obesity measured by children's and adolescents' body mass index (BMI) are leveling off in many high-income countries, albeit at a high plateau, while the prevalence has continued to increase in parts of Asia (for further details see also [33]). The excessive gain of body fat during childhood significantly increases the risk of adult obesity and is a major driver of the MetS since it impacts the development of cardio-metabolic complications such as hypertension, dyslipidemia, and impaired glucose metabolism [21].

Reisinger et al. [40] conducted a systemic review on the prevalence of pediatric MetS where 31 studies (published between 2014 and 2019) were included that used at least one of the following four classifications: International Diabetes Federation [17], Cook et al. [15], de Ferranti et al. [16], and Ford et al. [20] where the IDF definition gives the lowest prevalence values. Only, eight studies involved children below the age of ten. Thus, it is not surprising that a huge variation in prevalence values was observed with the lowest prevalence of 0.3% (IDF) in Colombian school children and the highest prevalence of 26.4% (de Ferranti et al.) in Iranian children and adolescents. A study on 2,149 children and adolescents aged 6–17 years in Saudi Arabia [5] may illustrate the huge differences in MetS prevalence according to the various criteria. Here, MetS prevalence was estimated as 2.0% according to IDF, 4.9% according to Cook et al. and Ford et al., and 17.5% according to de Ferranti et al. Having a look at some specific examples, it becomes obvious that there was some heterogeneity across the studies included not only with respect to the MetS criteria used but also with respect to sample sizes and age groups.

For instance, in a nationwide survey of 37,504 Brazilian adolescents aged 12–17 years, the prevalence of MetS was 2.6% (IDF) [26]. A Chinese survey including adults and adolescents reported a prevalence of 3.8% (Cook et al.) based on 11,174 adolescents aged 10–17 years [47] which is comparable to the MetS prevalence of 3% reported in a survey in Beijing, conducted in 2017, with 1766 adolescents aged 10–15 years [48]. Based on data from the 2010–2012 Korea National Health and Nutrition Examination Survey-V, a MetS prevalence of 5.7% (Ford et al.) or 2.1% (IDF), respectively, was reported for 2330 children aged 10–18 years [25]. In a sample of 4641 Iranian children and adolescents aged 10–18 years, a MetS prevalence of 3.6% (IDF) was observed for boys and of 5.9% for girls [6]. The MetS prevalence among US adolescents [41] was estimated as 5.4% based on a sample of 1623 adolescents aged 12–19 years from the National Health and Nutrition Examination Survey 2005–2012.

In a more recent systematic review by Noubiap et al. [37], 169 studies including 550,405 children and adolescents from 44 countries were pooled, with the majority of data points from adolescents. An adapted version of the criteria according to Ford et al. was used to assess the MetS prevalence where the overall MetS prevalence in 2020 was estimated as 2.8% (4.8%) among children (adolescents). The studies were further summarized into various categories. Looking for instance at income, the MetS for children (adolescents in brackets) was estimated as 2.2% (5.5%) in high-income countries, 3.1% (3.9%) in upper-middle-income countries, 2.6% (4.5%) in lower-middle-income countries, and 3.5% (7.0%) in low-income countries. Thus, in general, the MetS prevalence was higher among adolescents compared to children, although there have been some exceptions on the regional level. Looking at the regional level (for a description on country level, see Noubiap et al. 2022), the MetS prevalence for children (adolescents in brackets) in Central and Eastern Europe was estimated at 2.3% (4.4%); Northwestern Europe 1.4% (3.7%); Southwestern Europe 2.0% (5.2%); Central Asia, Middle East, and North Africa 3.4% (5.7%); East and Southeast Asia 2.2% (3.7%); South Asia 2.5% (3.9%); high-income Asia Pacific 2.0% (5.2%); high-income English speaking countries 2.5% (6.7%); Latin America and the Caribbean 5.6% (5.4%); Oceania 3.0% (5.7%); and Sub-Saharan Africa 3.0% (6.2%).

In the following, we will consider a pan-European cohort study in more detail in particular with respect to the development of MetS during the transitional phase from childhood to adolescence and to potential risk factors.

### The IDEFICS/I.Family cohort

The IDEFICS/I.Family cohort was set up as a pan-European multi-center population-based study to identify risk factors for diet- and lifestyle-related diseases, with a focus on childhood overweight and metabolic disorders. About 2,000 children were recruited at baseline in kindergartens and schools in each of the eight participating European countries (Belgium, Cyprus, Estonia, Germany, Hungary, Italy, Spain, Sweden). This resulted in 16,229 children aged 2–9.9 years who took part in the baseline survey (*W*_0_) that was conducted in 2007/2008. The extensive examination program of W_0_ was repeated in 2009/2010 (*W*_1_). In this 1st follow-up, 13,596 children aged 2–12 years participated. For the 2nd follow-up (2013/2014; *W*_2_), siblings were also invited to participate. Thus, in total 9617 children aged 5–17 years were examined. The 3rd follow-up was conducted during the corona pandemic (2021/2022; *W*_3_) as online survey where 5322 children, adolescents, and young adults aged 11–26 years filled in the questionnaires. For further details on the IDEFICS/I.Family cohort, refer to [3]. In 2012, Poland joined this cohort as a ninth cohort center.

#### Examination protocol

All participants passed an exhaustive, highly standardized examination protocol. Questionnaires were filled by parents or by adolescents themselves to learn about the socio-demographic background of the families, the medical history of the children and their lifestyle factors. Dietary behavior was assessed via a children´s eating habits questionnaire comprising a food frequency module and 24-h recalls. In addition, school meals were weighed before and after food intake. Physical activity was measured in a subgroup via accelerometry whereas physical fitness was assessed via a whole test battery. Anthropometric measurements such as height, weight, skinfold thickness, hip, waist, and neck circumferences were taken using the same measurement instruments in all centers. In addition, biological samples were collected and stored at −80 °C (blood serum/plasma) and −20 °C (urine and saliva), respectively.

All examination modules and survey instruments are described in detail in [7]. The study was approved by the local ethics committees, and informed consent was obtained from the children (oral consent) and their parents (written consent) for each examination module separately. The IDEFICS/I.Family cohort study is registered in the ISRCTN clinical trial registry (https://www.isrctn.com/ISRCTN62310987).

#### Metabolic syndrome

A broad spectrum of associations between obesity and potential risk or protective factors, respectively, has been investigated cross-sectionally and longitudinally (see below). However, the assessment of clinical parameters in children is hampered by the lack of appropriate, risk-based cut-off values that define elevated levels in relation to health outcomes. In this situation it is common practice to use a statistical definition of elevated levels in relation to the distribution of the respective clinical parameters, such that upper and lower percentiles define the cut-offs beyond which parameter values are considered as being outside the “normal” range. Given the lack of appropriate data for such definitions in children, the IDEFICS/I.Family study offered a unique opportunity to calculate age- and sex-specific (and also height-specific in the case of blood pressure) reference values for numerous medical parameters based on the huge population-based dataset of 18,745 children [1]. In particular, a new definition of the MetS was derived tailored to young children aged 2–11 years [2] that has been suggested for worldwide use in a comment published in the Lancet Child & Adolescent Health [13]. By not using adult criteria this suggestion overcomes several of the limitations of former definitions of the MetS in children. Applying adult criteria to children would ignore growth- and sex-related changes in body composition and physiology, of which the obesity rebound is a well-known example. As the four components of the MetS, (1) waist circumference to assess obesity, (2) systolic (SBP) and diastolic blood pressure (DBP) to assess hypertension, (3) triglycerides or high-density lipoprotein cholesterol (HDL-C) to assess dyslipidemia and (4) HOMA-insulin resistance or fasting blood glucose to assess insulin sensitivity are recommended. As cut-offs the 90th (P90) or 95th percentiles (P95) are proposed where a child should be monitored if the 90th percentiles of at least three of the components are exceeded and actions should be taken if the 95th percentiles of at least three of the components are exceeded (see Table 1).

**Table 1:** Definition of pediatric metabolic syndrome

Excess adiposity WAIST +	Blood pressure BP +	Blood lipids LIPIDS +	Blood glucose/insulin GLU +
Waist circumference ≥ P90/P95	SBP or DBP ≥ P90/P95	Triglycerides ≥ P90/P95 or HDL-C ≤ P10/P5	HOMA-insulin resistance ≥ P90/P95 or fasting glucose ≥ P90/P95

*HOMA*, homeostasis model assessment

According to these criteria, 1.5% of normal weight or thin children of the IDEFICS cohort, 14.1% of overweight children, 31.5% of obese children and 5.5% of all children needed to be at least monitored with only minor differences in prevalence between boys and girls (children’s weight status classified according to [14]).

**Fig. 1:**
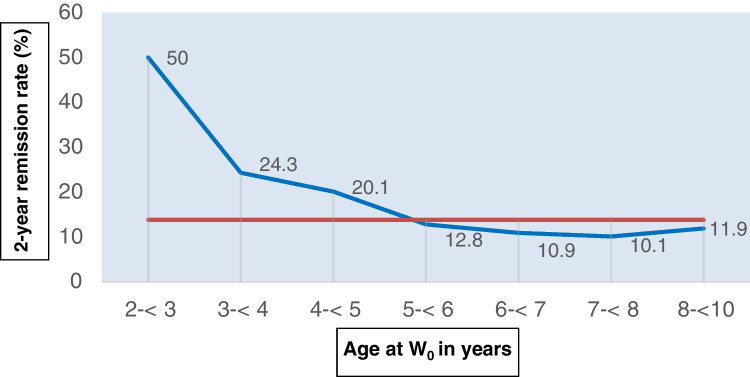
Remission rates of overweight/obesity for children of the IDEFICS cohort between baseline and first follow-up (red line: weighted average remission rate over all age groups; blue line: age-specific remission rate)

Since it is well known that childhood obesity tracks into adulthood the dynamics of metabolic status during the growth period of children was of major interest. In a first step, the spontaneous remission rate of overweight and obesity (defined by age-specific BMI cut-offs) between the baseline survey *W*_0_ and the first follow-up examination *W*_1_ was investigated in 13,596 children, 1.9 to 2 years after the baseline examination. As it can be clearly seen in Fig. 1, children aged 2–3 years have a 50% remission rate which declines to 24.3% in 3- to 4-year-old children and further down to 10.1% in children aged 7–8 years. These results indicate that the variability of weight status decreases dramatically already in the early years of life until the child reaches school age.

As a major finding of a further prospective analysis of weight status and rate of BMI change in these children, we showed a positive association of current BMI and later MetS score. Moreover, the rate of BMI change was strongly and positively associated with the MetS score and this association was strongest for the rate of BMI change during early childhood. The data indicate that the time window between 9 months to 6 years of age is the most sensitive period during which a rapid increase in BMI subsequently is followed by an elevated metabolic risk in children [8].

**Fig. 2:**
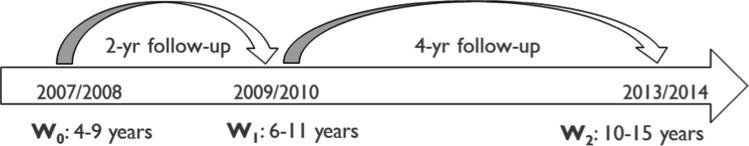
Temporal sequence of the first three examination waves of the IDEFICS/I.Family cohort

In a next step, Börnhorst et al. [9] investigated the metabolic status of our IDEFICS children during their transition into adolescence. The analysis included 6768 children aged 4–15 years in whom waist circumference, blood pressure, blood glucose, and lipids were measured at least two times during the first three examination waves of the IDEFICS/I.Family cohort. Children with blood measurements taken in non-fasting status and children, who used any medications at time of measurements, were excluded from the analysis. Since multiple combinations and change patterns could occur over time for the four components of the MetS (each with three levels: normal, ≥ P90, ≥ P95), the so-called latent transition analysis was applied to identify latent groups of children with similar metabolic profiles and to estimate the transition probabilities over time (Fig. 2).

**Fig. 3:**
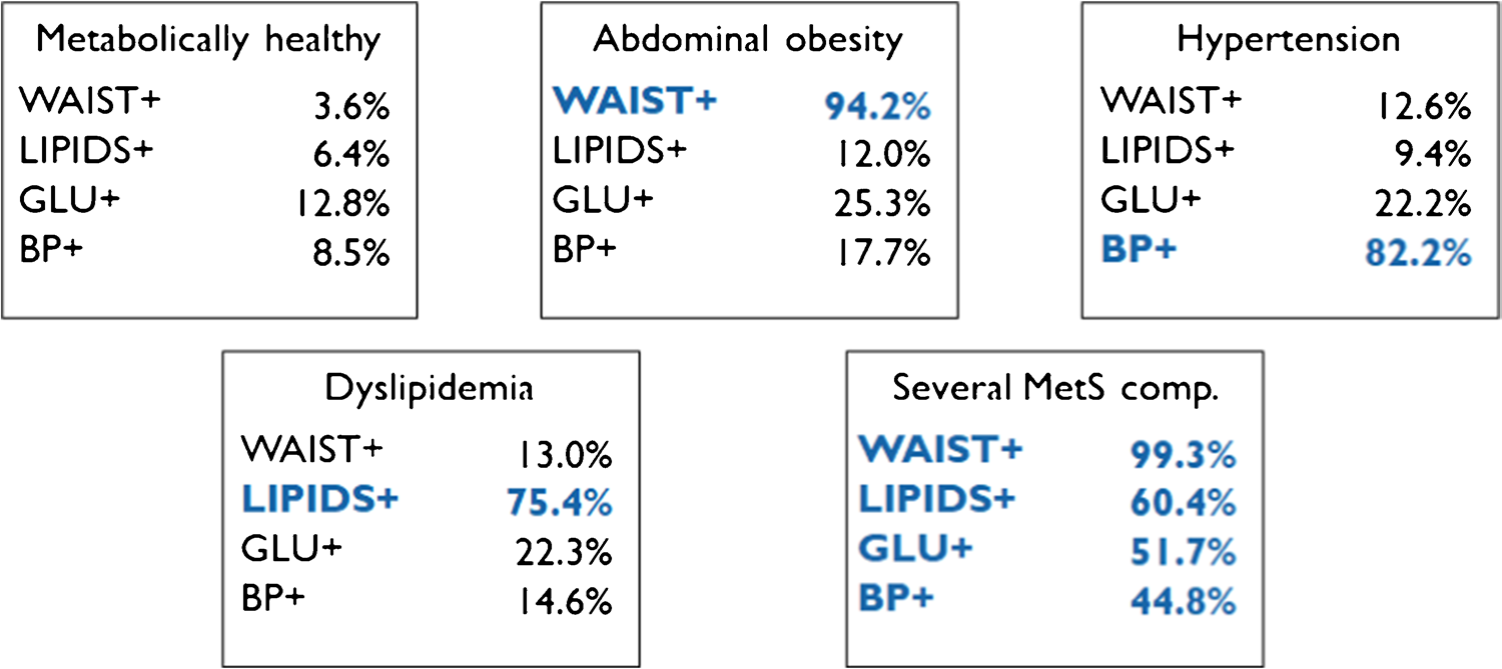
Five latent groups identified by latent transition analysis where percentages represent the estimated probabilities of a child having elevated values (≥ P90)

The best model fit according to the Bayesian Information criterion (BIC) was achieved for five groups as defined in Fig. 3, where no group was characterized by elevated fasting glucose as the dominant trait. Children who were classified as metabolically healthy at W_0_ stayed metabolically healthy at W_1_ with a probability of 86.6%. The vast majority of children who were assigned to the group “Abdominal obesity” stayed in this group (79.3%) while 18.5% even switched to the group “Several MetS comp.” The most striking result was observed for children being assigned to the “Several MetS comp” group: these children stayed in this group at W_1_ with a probability 99.8%. Looking at these children further, the probability of staying in this group at *W*_2_ was estimated as 88.3%. With a probability of 7.3% and 3.5%, respectively, children belonging to this group at *W*_1_ moved to the “abdominal obesity” and the “dyslipidemia” groups at *W*_2_. Children moved from the “several MetS comp” group to the metabolically healthy group at *W*_2_ with a probability of less than 1% which is a critical finding: recovery seems to be unlikely.

#### Major risk factors

According to Börnhorst et al. [10] and based on the patterns derived above, multivariate mixed-effects models were used to assess the age-dependent associations of modifiable and non-modifiable risk factors as well as C-reactive protein (CRP), with the log-transformed probabilities of developing abdominal obesity, hypertension, dyslipidemia, or several metabolic disturbances relative to the probability of staying metabolically healthy, with the latter serving as the reference group.

Having at least one media device in the child’s bedroom and not being member in a sports club increased the risk of having several elevated components of the MetS where the latter also increased the risk for dyslipidemia alone. A protective effect on abdominal obesity could be observed for children with better well-being. Surprisingly, no associations were found for breastfeeding duration, fruit and vegetable, or processed food consumption with any of the metabolic outcomes during the transition between childhood and adolescence.

Besides these modifiable risk factors, it was also observed that a high maternal body mass index and a family history of hypertension, both, were positively associated with the risk of abdominal obesity, dyslipidemia, hypertension, and elevated levels of several components of the MetS. A low or medium parental educational level of parents assessed by the International Standard Classification of Education (ISCED; [45]) as well as entering puberty at an early age were positively associated with the risk of abdominal obesity and of having several elevated components of the MetS.

In addition, higher CRP *z*-scores were positively associated with the risk of abdominal obesity, dyslipidemia, hypertension and of showing several elevated components of the MetS.

In a recent publication, Nagrani et al. [29] investigated the potential influence of environmental factors on the risk of prediabetes and MetS based on data from the IDEFICS/I.Family cohort. In this first analysis, Nagrani et al. focused on exposure to black carbon (BC) where the amount of BC particles in the children´s urine was used as measure of BC exposure as precise as possible. In a subgroup of 5,453 children, the annual exposure to ambient air pollutants at the place of residence was assessed using land use regression models and calculated the residential distance to major roads. Here, a positive association (1) between living not more than 250 m away from a major road and urinary black carbon levels compared to children living more than 250 m away from a major road and (2) between the concentration of log-transformed black carbon nano-particles in urine and MetS was observed.

## The epidemiology of the metabolic syndrome in adults

According to the WHO definition, MetS in adults is defined by abdominal obesity, hypertension, hyperlipidemia, and insulin resistance (for details see [23]). However, the various definitions of the MetS in adults differ with regard to the measured parameters and the specification of the cut-offs for each of its components. Depending on the definition used the prevalence estimates of MetS can vary substantially [36]. The prevalence of the MetS reported below is most often based on the International Diabetes Federation (IDF) criteria, but other definitions are also quite common as, e.g., the National Cholesterol Education Programme’s Adult Treatment Panel III criteria (NCEP ATP III) and according modifications of IDF and NCEP ATP III to account for Asian populations in particular for BMI and waist circumference as well as the American Heart Association/National Heart, Lung and Blood Institute (AHA/NLHBI) criteria.

### The global prevalence of metabolic syndrome and its components

Based on an analysis of 1698 population-based data sources with more than 19.2 million persons being at least 18 years old [30], it becomes obvious that the prevalence of obesity has significantly increased worldwide over the last decades. Age-standardized mean body mass index (BMI) increased globally from 21.7 kg/m^2^ in 1975 to 24.2 kg/m^2^ in 2014 in men, and from 22.1 kg/m^2^ to 24.4 kg/m^2^ in women.

With regard to hypertension, the NCD Risk Factor Collaboration [35] analyzed data on measured blood pressure and data on blood pressure treatment from 104 million persons aged 30–79 years collected in 1201 population-representative studies carried out from 1990 to 2019. Hypertension was defined as having systolic blood pressure 140 mm Hg or greater, diastolic blood pressure 90 mm Hg or greater, or taking medication for hypertension. Although the number of people with hypertension doubled from 1990 to 2019, the age-standardized prevalence remained nearly unchanged (1990: 32% in women and men; 2019: 32% in women and 34% in men). This is mainly due to a decrease in high-income countries and an increase in some low- and middle-income countries.

The NCD Risk Factor Collaboration [34] also investigated worldwide trends in mean total, non-HDL and HDL cholesterol levels for 200 countries. For this purpose, data from 1,127 population-based studies collected in 102.6 million individuals aged 18 years and older were used. There were nearly no changes in global age-standardized mean non-HDL cholesterol from 1980 to 2018 where a substantial decrease in non-HDL cholesterol in high-income western regions and central and eastern Europe was compensated by an increase in east and southeast Asia, parts of sub-Saharan Africa and Melanesia. Global age-standardized mean HDL cholesterol remained stable for women whereas a slight decrease could be observed for men. The authors concluded that there has been a “major global repositioning of lipid-related risk,” where non-optimal cholesterol patterns, in particular for non-HDL cholesterol, shifted from high-income countries in northwestern Europe, north America, and Australasia to middle-income countries in east and southeast Asia, as well as to some countries in Oceania and central Latin America.

Based on pooled data on diabetes via measured biomarkers from 751 population-based studies with in total 4,372,000 adults, the NCD Risk Factor Collaboration [31] estimated worldwide trends in diabetes prevalence from 1980 to 2014. Diabetes was defined as fasting plasma glucose of at least 7.0 mmol/l, self-reported diabetes diagnosis, or use of insulin or oral hypoglycemic drugs. Age-standardized prevalence of diabetes increased globally: from 4.3% in 1980 to 9.0% in 2014 in men, and from 5.0 to 7.9% in women over the same time period. This resulted in an increase of adults with diabetes worldwide from 108 to 422 million. According to the estimates of the NCD Risk Factor Collaboration 28.5% of this increase was due to the rise in prevalence, 39.7% due to population growth and aging, and 31.8% due to the interaction of these factors. The temporal trend in the prevalence of type 2 diabetes was also confirmed by the recent Global Burden of Disease Study 2019 [42].

According to [43], the “incidence of MetS often parallels the incidence of obesity and incidence of type 2 diabetes.” Thus, it is not surprising that also the prevalence of the MetS is on the rise. For instance, according to the National Health and Nutrition Examination Survey (NHANES), the prevalence of MetS among US adults aged 18 years or older increased from 25.3 to 34.2% from 1988–1994 to 2007–2012 [28], where the MetS was defined according to the Joint Interim Statement (JIS) agreed upon by several medical associations as, e.g., IDF [4]. A recent update [27] including 8,183 non-pregnant NHANES participants aged 20 years or older reported a further increase: the prevalence of the MetS increased from 37.6% in 2011–2012 to 41.8% in 2017–2018.

A systematic review on the prevalence of the MetS among adults in the Asia–Pacific region [39] showed a huge between-country variation with a prevalence of 11.9% (NCEP ATP III) observed in a national survey of the Philippines in 2003 to 49.0% (modified NCEP ATP III) (34.8% according to IDF) in urban Pakistan (Karachi) in 2004 and 37.1% (IDF) in Malaysia in 2008. In most countries, the reported prevalence was higher in women than in men. For three countries time trends were reported, all showing an increasing prevalence among men and women: According to the Korean National Health and Nutrition Examination Survey (KNHANES) the prevalence in South Korea increased from 24.9% in 1998 to 31.3% (modified NCEP ATP III) in 2007. The prevalence of the MetS in Taiwan increased from 13.6% in a national survey during 1993–1996 to 25.5% (modified NCEP ATP III) in the 2005–2008 survey. In China, the prevalence increased from 13.7% in 2000/01 to 21.3% (NCEP ATP III) in 2009.

Hiramatsu et al. [22] recently reported time trends of the prevalence in Japanese men (69,643) and women (92,092) aged 40–75 years using a 6-year follow-up from 2012 to 2018 of the population covered by the Shizuoka Kokuho Database. Between 2012 and 2017 the prevalence according to the Joint Interim Statement (JIS) criteria increased from 29.2 to 35.9% in men and from 33.1 to 42.5% in women.

In a recent African review [12], 345 studies from 29 African countries were included with in total 156,464 participants recruited between 1985 and 2021. The largest number of studies was contributed by Nigeria (66), followed by South Africa (49), Ethiopia (36), Ghana (34), Egypt (31), Tunisia (29), Morocco (18), and Cameroon (13). Again various definitions of the MetS were used to calculate the prevalence. The authors calculated a pooled prevalence of 32.4%. Based on 151 studies that reported sex-specific prevalence, the pooled prevalence of MetS was significantly higher in African women (36.9%) than in men (26.7%). Comparing geographic regions, there were no major differences with a pooled prevalence of 33.6% in South Africa (55 studies), 33.1% in West Africa (112 studies), 32.8% in North Africa (94 studies), 30.3% in East Africa (61 studies) and 30.1% in Central Africa (23 studies). However, major differences in prevalence estimates were observed among individual countries with Algeria having the highest prevalence (43.9%) and Sudan the lowest (1.5%).

Recently, a global meta-analysis including 28 million individuals analyzed the geographic distribution of MetS and its components [36]. The meta-analysis summarizes 1129 data records from 663 articles published until 2021 to provide a global overview and to compare the prevalence of MetS and its components by geographic region and socio-economic levels while applying 11 different definitions and cut-offs. When the authors compared the global prevalence by definition of MetS they observed a huge variation ranging from 12.5% according to ATP III (BMI ≥ 25 kg/m^2^) to 28.2% according to IDF and 31.4% according to JIS. The prevalence increased with age and study year, was higher in urban than rural areas and was generally higher in women as compared to men. The prevalence appeared to increase by income level of a country and was significantly higher in the Eastern Mediterranean Region (34.6%; IDF definition) and Americas (33.4%; IDF definition) as compared to other regions of the world. The prevalence was lowest in Africa (23.1%; IDF definition). According to the IDF definition the prevalence for the WHO region Europe was estimated at 31.5%.

### The NAKO Health Study

To better understand the development of the MetS and its sub-clinical stages in adults, large population-based cohorts are indispensable, in particular, if these are multi-purpose cohorts that consider a wide range of potential exposures and outcomes and thus deliver valuable data on incidence and multiple determinants of diseases. In Europe, there are four major cohorts currently running: (1) The UK Biobank with 500,000 participants aged 40–69 years, (2) Constances in France with 219,000 participants aged 18–69 years, (3) Lifelines in The Netherlands with 15,000 participants aged 0–18 years, 140,000 aged 18–65 years, and 12,000 with 65 + years of age, (4) the NAKO Health Study in Germany with 205,000 participants aged 20–69 years. These cohorts will offer new knowledge allowing early detection and targeted prevention on a population level.

In the following, we will present some recent results based on data from the first 100,000 participants of the NAKO Health Study [38], which is the largest population-based prospective study in Germany so far. It aims at identifying the determinants and risk factors of chronic diseases in relation to lifestyle, environmental, genetic, and socio-demographic factors and at investigating the impact of geographic and socio-economic differences on the health status of the German adult population. The baseline examination took place from 2014 to 2019, the first follow-up examination will run until spring 2024 and a subsequent second follow-up examination is planned for 2024 to 2029. The exhaustive examination program covers an in-person interview on the medical history and medications; self-completion questionnaires on health-related behaviors and socio-demographic and psychosocial factors; neuro-cognitive tests on attention, concentration, and memory; measurements of body height/weight, waist/hip circumference, body composition, abdominal fat, sugar metabolism, grip strength, physical fitness, physical activity (via accelerometer), blood pressure, heart rate, and cardiovascular parameters such as 3D-echocardiography, resting electrocardiogram (ECG), long-term ECG, sleep characteristic; analysis of exhaled air and assessment of lung function; assessment of dental health; collection of blood, saliva, nasal mucosa, urine, and stool. In addition, participants are asked for informed consent to link their primary data with secondary data sources like cancer registries and health insurances. More than 90% of the NAKO participants gave their consent for such a record linkage at baseline.

The percentage of overweight (obesity in brackets) among the first 100,000 NAKO participants was 46.2% (23.5%) in men compared to 29.7% (21.2%) in women [18]. There was a huge difference among age groups. For men, the prevalence of overweight (obesity in brackets) ranged from 30.3% (9.8%) in the age group 20–29 years to 49.0% (30.2%) or 51.7% (27.9%) in the 60–69 years old or men being at least 70 years, respectively. For women, the prevalence of overweight (obesity) ranged from 18.5% (9.2%) in the age group 20–29 years to 38.6% (29.3%) in those being at least 70 years. Comparing the distribution, women had a higher subcutaneous adipose tissue layer thickness compared to men whereas the average visceral adipose tissue layer thickness was higher in men across all groups with a clear positive age trend in both sexes.

According to [24], the percentage of a self-reported medical diagnosis of a metabolic disease in NAKO participants heavily varied between men and women and across age groups with, e.g., 55% of women reporting a first diagnosis of thyroid disease already below the age of 40 years. Table 2 shows a high prevalence of metabolic diseases in NAKO participants with unadjusted frequencies reaching 40.5% in men and 50.1% in women having at least one of them. The most frequent disease among men was hyperlipidemia (28.6%) while thyroid disease was most the most frequent disease among women (34.3%).

**Table 2:** Frequencies of one or more self-reported medical diagnoses of metabolic diseases by sex (adapted from [24])

	Men (n = 47,266)			Women (n = 54,540)		
	n	% (crude)	% (stand.*)	n	% (crude)	% (stand.*)
Diabetes mellitus	3,838	8.1	5.6	3,163	5.8	4.9
Hyperlipidemia	13,519	28.6	21.4	13,350	24.5	19.4
Gout	3,711	7.9	5.6	1,290	2.4	1.9
Thyroid disease	4,779	10.1	8.3	18.706	34.3	29.2
At least one of these diseases	19,157	40.5	31.5	27,298	50.1	27.5

*Direct age standardization based on Federal Statistical Office of Germany 2011

## Discussion and conclusions

Pooling studies have proven valuable when assessing the global distributions of MetS and their temporal trends as well in investigating the components and determinants of MetS. Of course, pooling studies also have limitations because different studies entail different variables and vary with regard to the depth of information which makes it difficult to investigate subgroups, for example. It is expected that the increasing acceptance of the FAIR (findable, accessible, interoperable, re-usable) guiding principles for data sharing [46] will enable future pooling studies to generate more comprehensive results.

The pooling studies described above show a dramatic global increase in obesity and metabolic syndrome over the last decades, reaching a prevalence of 28.2% when the IDF definition was applied, with substantial variation across countries. The prevalence was positively associated with the income level of a country and was higher in the Eastern Mediterranean Region and the Americas as compared to other regions of the world. The prevalence was lowest in Africa. In most countries the prevalence of MetS is higher in women compared to men and higher in urban as compared to rural areas. Regarding the components of the MetS the global picture is complex. The number of people with hypertension doubled from 1990 to 2019 but the age-standardized prevalence remained nearly unchanged. This is mainly due to a decrease in high-income countries and an increase in some low- and middle-income countries. Overall the age-standardized mean of non-HDL cholesterol levels did not change from 1980 to 2018 but this apparently masked a global geographic shift of the prevalence of non-optimal cholesterol patterns from high-income western regions to middle-income countries in east and southeast Asia, as well as to some countries in Oceania and central Latin America. The age-standardized prevalence of diabetes doubled between 1980 and 2014 reaching values of 9.0% in men and 7.9% in women globally.

Cohort studies like the NAKO Health Study or the IDEFICS/I.Family study are indispensable resources to monitor time trends and to capture major changes in health outcomes and determinants over the life-course. However, the results of the NAKO Health Study for diabetes and dyslipidemia are yet based on self-reports of study participants while the more valid biomarker-based data are still waiting to be published. Despite these limitations, the NAKO Health Study again showed a worrying high prevalence of metabolic disorders in the population.

According to the above analyses, about 40% of children in the considered age range showed metabolic disorders without major sex differences, where their prevalence increased with age, particularly in puberty. Dyslipidemia seems to be the most reversible component of the MetS in youth. However, dyslipidemia or hypertension rarely occurred in isolation but were more likely to manifest as a comorbid condition of abdominal obesity which seemed to be the starting point of further metabolic disturbances. As a most alarming result, we observed that nearly no child having several elevated components of the MetS returned to a metabolically healthy status during 6 years of follow-up. This is a clear indication of the need for early and —based on our results [11]— also for multi-level interventions where high-risk groups, i.e., those with low socio-economic status or maternal obesity may benefit most from well-targeted public health and education policies.

We should note that our use of a statistical definition of elevated clinical parameters where cut-offs are defined by percentile values is not ideal for two reasons: (1) such cut-offs depend on the distribution of the parameters of interest in the population from which they are derived and may thus differ between populations and (2) distributions shift over time with an increasingly obese global population and this may result in an inflation of reference values, if cut-offs are updated based on actual distributions. It is thus desirable to eventually replace such relative reference values by stringent absolute cut-offs that are clinically relevant, i.e., that are based on associated risks of developing well-defined disease outcomes.

## Declarations

**IDEFICS/I.Family Cohort**

### Availability of data and materials

The use of data of the IDEFICS/I.Family cohort requires a mutual agreement between the study consortia and interested third parties on a case-by-case basis. Corresponding requests can be sent to the study coordinator: i.family@leibniz-bips.de.

**NAKO**

### Availability of data and materials

Access to and use of NAKO data and biosamples can be obtained via an electronic application portal (https://transfer.nako.de).

## References

[1] Ahrens W, Moreno LA, Pigeot I (2014) Filling the gap: international reference values for health care in children. Int J Obes (Lond) 38(Suppl 2):S2-3. 10.1038/ijo.2014.129 25219409 10.1038/ijo.2014.129 PMC7420737

[2] Ahrens W, Moreno LA, Mårild S, Molnár D, Siani A, De Henauw S, Böhmann J, Günther K, Hadjigeorgiou C, Iacoviello L, Lissner L, Veidebaum T, Pohlabeln H, Pigeot I, IDEFICS consortium (2014) Metabolic syndrome in young children: definitions and results of the IDEFICS study. Int J Obes (Lond) 38(Suppl 2):S4-14. 10.1038/ijo.2014.130 25376220 10.1038/ijo.2014.130

[3] Ahrens W, Siani A, Adan R, De Henauw S, Eiben G, Gwozdz W, Hebestreit A, Hunsberger M, Kaprio J, Krogh V, Lissner L, Molnár D, Moreno LA, Page A, Picó C, Reisch L, Smith RM, Tornaritis M, Veidebaum T, Williams G, Pohlabeln H, Pigeot I (2017) Cohort Profile: The transition from childhood to adolescence in European children-how I.Family extends the IDEFICS cohort. Int J Epidemiol 46:1394–1395j. 10.1093/ije/dyw317 10.1093/ije/dyw317 PMC5837508 28040744

[4] Alberti KG, Eckel RH, Grundy SM, Zimmet PZ, Cleeman JI, Donato KA, Fruchart JC, James WP, Loria CM, Smith Jr SC 2009 Harmonizing the metabolic syndrome: a joint interim statement of the International Diabetes Federation Task Force on Epidemiology and Prevention; National Heart, Lung, and Blood Institute; American Heart Association; World Heart Federation; International Atherosclerosis Society; and International Association for the Study of Obesity Circulation 120 1640 1645 10.1161/CIRCULATIONAHA.109.192644 10.1161/CIRCULATIONAHA.109.192644 19805654

[5] Al-Hussein FA, Tamimi W, Al Banyan E, Al-Twaijri YA, Tamim H (2014) Cardiometabolic risk among Saudi children and adolescents: Saudi childrens overweight, obesity, and lifestyles (S.Ch.O.O.Ls) study. Ann Saudi Med 34:46–53. 10.5144/0256-4947.2014.46 24658553 10.5144/0256-4947.2014.46 PMC6074937

[6] Ataie-Jafari A, Heshmat R, Kelishadi R, Ardalan G, Mahmoudarabi M, Rezapoor A, Motlagh ME, Asayesh H, Larijani B, Qorbani M (2014) Generalized or abdominal obesity: which one better identifies cardiometabolic risk factors among children and adolescents? The CASPIAN III study. J Trop Pediatr 60:377–385. 10.1093/tropej/fmu033 25037734 10.1093/tropej/fmu033

[7] Bammann K, Lissner L, Pigeot I, Ahrens W (2019) Instruments for health surveys in children and adolescents. Springer International Publishing, Cham

[8] Börnhorst C, Tilling K, Russo P, Kourides Y, Michels N, Molnár D, Rodríguez G, Moreno LA, Krogh V, Ben-Shlomo Y, Ahrens W, Pigeot I (2016) Associations between early body mass index trajectories and later metabolic risk factors in European children: the IDEFICS study. Eur J Epidemiol 31:513–525. 10.1007/s10654-015-0080-z 26297214 10.1007/s10654-015-0080-z

[9] Börnhorst C, Russo P, Veidebaum T, Tornaritis M, Molnár D, Lissner L, Marild S, De Henauw S, Moreno LA, Intemann T, Wolters M, Ahrens W, Floegel A (2019) Metabolic status in children and its transitions during childhood and adolescence-the IDEFICS/I.Family study. Int J Epidemiol 48:1673–1683. 10.1093/ije/dyz097 31098634 10.1093/ije/dyz097

[10] Börnhorst C, Russo P, Veidebaum T, Tornaritis M, Molnár D, Lissner L, Mårild S, De Henauw S, Moreno LA, Floegel A, Ahrens W, Wolters M (2020) The role of lifestyle and non-modifiable risk factors in the development of metabolic disturbances from childhood to adolescence. Int J Obes (Lond) 44:2236–2245. 10.1038/s41366-020-00671-8 32943762 10.1038/s41366-020-00671-8 PMC7577850

[11] Börnhorst C, Pigeot I, De Henauw S, Formisano A, Lissner L, Molnár D, Moreno LA, Tornaritis M, Veidebaum T, Vrijkotte T, Didelez V (2023) The effects of hypothetical behavioral interventions on the 13-year incidence of overweight/obesity in children and adolescents. Int J Behav Nutr Phys Act 20(1):100. 10.1186/s12966-023-01501-6 37620898 10.1186/s12966-023-01501-6 PMC10463721

[12] Bowo-Ngandji A, Kenmoe S, Ebogo-Belobo JT, Kenfack-Momo R, Takuissu GR, Kengne-Ndé C, Mbaga DS, Tchatchouang S, Kenfack-Zanguim J, Lontuo Fogang R, Zeuko’o Menkem E, Ndzie Ondigui JL, Kame-Ngasse GI, Magoudjou-Pekam JN, Wandji Nguedjo M, Assam Assam JP, Enyegue Mandob D, Ngondi JL (2023) Prevalence of the metabolic syndrome in African populations: a systematic review and meta-analysis. PLoS ONE 18:e0289155. 10.1371/journal.pone.0289155 37498832 10.1371/journal.pone.0289155 PMC10374159

[13] Chiarelli F, Mohn A (2017) Early diagnosis of metabolic syndrome in children. Lancet Child Adolesc Health 1:86–88. 10.1016/S2352-4642(17)30043-3 30169210 10.1016/S2352-4642(17)30043-3

[14] Cole TJ, Lobstein T (2012) Extended international (IOTF) body mass index cut-offs for thinness, overweight and obesity. Pediatr Obes 7:284–894. 10.1111/j.2047-6310.2012.00064.x 22715120 10.1111/j.2047-6310.2012.00064.x

[15] Cook S, Weitzman M, Auinger P, Nguyen M, Dietz WH (2003) Prevalence of a metabolic syndrome phenotype in adolescents: findings from the third National Health and Nutrition Examination Survey, 1988–1994. Arch Pediatr Adolesc Med 157:821–827. 10.1001/archpedi.157.8.821 12912790 10.1001/archpedi.157.8.821

[16] de Ferranti SD, Gauvreau K, Ludwig DS, Neufeld EJ, Newburger JW, Rifai N (2004) Prevalence of the metabolic syndrome in American adolescents: findings from the Third National Health and Nutrition Examination Survey. Circulation 110:2494–2497. 10.1161/01.CIR.0000145117.40114.C7 15477412 10.1161/01.CIR.0000145117.40114.C7

[17] Expert Panel on Detection Evaluation, and Treatment of High Blood Cholesterol in Adults (2001) Executive summary of the third report of the National Cholesterol Education Program (NCEP) expert panel on detection, evaluation, and treatment of high blood cholesterol in adults (Adult Treatment Panel III). JAMA 285:2486–2497. 10.1001/jama.285.19.2486 11368702 10.1001/jama.285.19.2486

[18] Fischer B, Sedlmeier AM, Hartwig S, Schlett CL, Ahrens W, Bamberg F, Baurecht H, Becher H, Berger K, Binder H, Bohn B, Carr PR, Castell S, Franzke CW, Fricke J, Gastell S, Greiser KH, Günther K, Jaeschke L, Kaaks R, Kemmling Y, Krist L, Kuß O, Legath N, Lieb W, Linseisen J, Löffler M, Michels KB, Mikolajczyk R, Niedermaier T, Norman K, Obi N, Peters A, Pischon T, Schikowski T, Schipf S, Schmidt B, Schulze MB, Stang A, Stojicic J, Tiller D, Völzke H, Waniek S, Leitzmann MF (2020) Anthropometrische Messungen in der NAKO Gesundheitsstudie – mehr als nur Größe und Gewicht [Anthropometric measures in the German National Cohort-more than weight and height]. Bundesgesundheitsblatt Gesundheitsforschung Gesundheitsschutz 63:290–300. 10.1007/s00103-020-03096-w 10.1007/s00103-020-03096-w 32020361

[19] Ford ES, Li C (2008) Defining the metabolic syndrome in children and adolescents: will the real definition please stand up? J Pediatr 152:160–164. 10.1016/j.jpeds.2007.07.056 18206681 10.1016/j.jpeds.2007.07.056

[20] Ford ES, Ajani UA, Mokdad AH, Health N, Examination N (2005) The metabolic syndrome and concentrations of C-reactive protein among U.S. youth. Diabetes Care 28:878–881. 10.2337/diacare.28.4.878 15793189 10.2337/diacare.28.4.878

[21] Gregory JW (2019) Prevention of obesity and metabolic syndrome in children. Front Endocrinol 10:669. 10.3389/fendo.2019.00669 10.3389/fendo.2019.00669 PMC6779866 31632348

[22] Hiramatsu Y, Ide H, Furui Y (2023) Differences in the components of metabolic syndrome by age and sex: a cross-sectional and longitudinal analysis of a cohort of middle-aged and older Japanese adults. BMC Geriatr 23:438. 10.1186/s12877-023-04145-0 37460963 10.1186/s12877-023-04145-0 PMC10353138

[23] Huang PL (2009) A comprehensive definition for metabolic syndrome. Dis Model Mech 2:231–237. 10.1242/dmm.001180 19407331 10.1242/dmm.001180 PMC2675814

[24] Jaeschke L, Steinbrecher A, Greiser KH, Dörr M, Buck T, Linseisen J, Meisinger C, Ahrens W, Becher H, Berger K, Braun B, Brenner H, Castell S, Fischer B, Franzke CW, Gastell S, Günther K, Hoffmann W, Holleczek B, Jagodzinski A, Kaaks R, Kluttig A, Krause G, Krist L, Kuß O, Lehnich AT, Leitzmann M, Lieb W, Löffler M, Michels KB, Mikolajczyk R, Peters A, Schikowski T, Schipf S, Schmidt B, Schulze M, Völzke H, Willich SN, Pischon T (2020) Erfassung selbst berichteter kardiovaskulärer und metabolischer Erkrankungen in der NAKO Gesundheitsstudie: Methoden und erste Ergebnisse [Assessment of self-reported cardiovascular and metabolic diseases in the German National Cohort (GNC, NAKO Gesundheitsstudie): methods and initial results]. Bundesgesundheitsblatt Gesundheitsforschung Gesundheitsschutz 63:439–451. German. 10.1007/s00103-020-03108-9 10.1007/s00103-020-03108-9 32157352

[25] Kim S, So WY (2016) Prevalence of metabolic syndrome among Korean adolescents according to the National Cholesterol Education Program, Adult Treatment Panel III and International Diabetes Federation. Nutrients 8:588. 10.3390/nu8100588 27706073 10.3390/nu8100588 PMC5083976

[26] Kuschnir MC, Bloch KV, Szklo M, Klein CH, Barufaldi LA, Abreu Gde A, Schaan B, da Veiga GV, da Silva TL, de Vasconcellos MT, de Moraes AJ, Borges AL, de Oliveira AM, Tavares BM, de Oliveira CL, Cunha Cde F, Giannini DT, Belfort DR, Santos EL, de Leon EB, Fujimori E, Oliveira ER, Magliano Eda S, Vasconcelos Fde A, Azevedo GD, Brunken GS, Guimarães IC, Faria Neto JR, Oliveira JS, de Carvalho KM, Gonçalves LG, Monteiro MI, Santos MM, Muniz PT, Jardim PC, Ferreira PA, Montenegro RM Jr, Gurgel RQ, Vianna RP, Vasconcelos SM, Martins SM, Goldberg TB (2016) ERICA: prevalence of metabolic syndrome in Brazilian adolescents. Rev Saude Publica 50 Suppl 1(Suppl 1):11s. 10.1590/S01518-8787.2016050006701 10.1590/S01518-8787.2016050006701 PMC4767042 26910546

[27] Liang X, Or B, Tsoi MF, Cheung CL, Cheung BMY (2023) Prevalence of metabolic syndrome in the United States National Health and Nutrition Examination Survey 2011–18. Postgrad Med J 99:985–992. 10.1093/postmj/qgad008 36906842 10.1093/postmj/qgad008

[28] Moore JX, Chaudhary N, Akinyemiju T (2017) Metabolic syndrome prevalence by race/ethnicity and sex in the United States, National Health and Nutrition Examination Survey, 1988–2012. Prev Chronic Dis 14:E24. 10.5888/pcd14.160287 28301314 10.5888/pcd14.160287 PMC5364735

[29] Nagrani R, Marron M, Bongaerts E, Nawrot TS, Ameloot M, de Hoogh K, Vienneau D, Lequy E, Jacquemin B, Guenther K, De Ruyter T, Mehlig K, Molnár D, Moreno LA, Russo P, Veidebaum T, Ahrens W, Buck C, IDEFICS and I. Family consortia (2023) Association of urinary and ambient black carbon, and other ambient air pollutants with risk of prediabetes and metabolic syndrome in children and adolescents. Environ Pollut 317:120773. 10.1016/j.envpol.2022.120773 36455765 10.1016/j.envpol.2022.120773

[30] NCD Risk Factor Collaboration (NCD-RisC) (2016) Trends in adult body-mass index in 200 countries from 1975 to 2014: a pooled analysis of 1698 population-based measurement studies with 19·2 million participants. Lancet 387(10026):1377–1396. 10.1016/S0140-6736(16)30054-X. (**Erratum.In:Lancet.2016May14;387(10032):1998**) 27115820 10.1016/S0140-6736(16)30054-X PMC7615134

[31] NCD Risk Factor Collaboration (NCD-RisC) (2016) Worldwide trends in diabetes since 1980: a pooled analysis of 751 population-based studies with 4.4 million participants. Lancet 387(10027):1513–1530. 10.1016/S0140-6736(16)00618-8 27061677 10.1016/S0140-6736(16)00618-8 PMC5081106

[32] NCD Risk Factor Collaboration (NCD-RisC) (2017) Worldwide trends in body-mass index, underweight, overweight, and obesity from 1975 to 2016: a pooled analysis of 2416 population-based measurement studies in 128·9 million children, adolescents, and adults. Lancet 390:2627–2642. 10.1016/S0140-6736(17)32129-3 29029897 10.1016/S0140-6736(17)32129-3 PMC5735219

[33] NCD Risk Factor Collaboration (NCD-RisC) (2020) Height and body-mass index trajectories of school-aged children and adolescents from 1985 to 2019 in 200 countries and territories: a pooled analysis of 2181 population-based studies with 65 million participants. Lancet 396:1511–1524. 10.1016/S0140-6736(20)31859-6 33160572 10.1016/S0140-6736(20)31859-6 PMC7658740

[34] NCD Risk Factor Collaboration (NCD-RisC) (2020) Repositioning of the global epicentre of non-optimal cholesterol. Nature 582(7810):73–77. 10.1038/s41586-020-2338-1 32494083 10.1038/s41586-020-2338-1 PMC7332422

[35] NCD Risk Factor Collaboration (NCD-RisC) (2021) Worldwide trends in hypertension prevalence and progress in treatment and control from 1990 to 2019: a pooled analysis of 1201 population-representative studies with 104 million participants. Lancet 398(10304):957–980. 10.1016/S0140-6736(21)01330-1 34450083 10.1016/S0140-6736(21)01330-1 PMC8446938

[36] Noubiap JJ, Nansseu JR, Lontchi-Yimagou E, Nkeck JR, Nyaga UF, Ngouo AT, Tounouga DN, Tianyi FL, Foka AJ, Ndoadoumgue AL, Bigna JJ (2022) Geographic distribution of metabolic syndrome and its components in the general adult population: a meta-analysis of global data from 28 million individuals. Diabetes Res Clin Pract 188:109924. 10.1016/j.diabres.2022.109924 35584716 10.1016/j.diabres.2022.109924

[37] Noubiap JJ, Nansseu JR, Lontchi-Yimagou E, Nkeck JR, Nyaga UF, Ngouo AT, Tounouga DN, Tianyi FL, Foka AJ, Ndoadoumgue AL, Bigna JJ (2022) Global, regional, and country estimates of metabolic syndrome burden in children and adolescents in 2020: a systematic review and modelling analysis. Lancet Child Adolesc Health 6:158–170. 10.1016/S2352-4642(21)00374-6 35051409 10.1016/S2352-4642(21)00374-6

[38] Peters A; German National Cohort (NAKO) Consortium; Peters A, Greiser KH, Göttlicher S, Ahrens W, Albrecht M, Bamberg F, Bärnighausen T, Becher H, Berger K, Beule A, Boeing H, Bohn B, Bohnert K, Braun B, Brenner H, Bülow R, Castell S, Damms-Machado A, Dörr M, Ebert N, Ecker M, Emmel C, Fischer B, Franzke CW, Gastell S, Giani G, Günther M, Günther K, Günther KP, Haerting J, Haug U, Heid IM, Heier M, Heinemeyer D, Hendel T, Herbolsheimer F, Hirsch J, Hoffmann W, Holleczek B, Hölling H, Hörlein A, Jöckel KH, Kaaks R, Karch A, Karrasch S, Kartschmit N, Kauczor HU, Keil T, Kemmling Y, Klee B, Klüppelholz B, Kluttig A, Kofink L, Köttgen A, Kraft D, Krause G, Kretz L, Krist L, Kühnisch J, Kuß O, Legath N, Lehnich AT, Leitzmann M, Lieb W, Linseisen J, Loeffler M, Macdonald A, Maier-Hein KH, Mangold N, Meinke-Franze C, Meisinger C, Melzer J, Mergarten B, Michels KB, Mikolajczyk R, Moebus S, Mueller U, Nauck M, Niendorf T, Nikolaou K, Obi N, Ostrzinski S, Panreck L, Pigeot I, Pischon T, Pschibul-Thamm I, Rathmann W, Reineke A, Roloff S, Rujescu D, Rupf S, Sander O, Schikowski T, Schipf S, Schirmacher P, Schlett CL, Schmidt B, Schmidt G, Schmidt M, Schöne G, Schulz H, Schulze MB, Schweig A, Sedlmeier AM, Selder S, Six-Merker J, Sowade R, Stang A, Stegle O, Steindorf K, Stübs G, Swart E, Teismann H, Thiele I, Thierry S, Ueffing M, Völzke H, Waniek S, Weber A, Werner N, Wichmann HE, Willich SN, Wirkner K, Wolf K, Wolff R, Zeeb H, Zinkhan M, Zschocke J (2022) Framework and baseline examination of the German National Cohort (NAKO). Eur J Epidemiol 37:1107–1124. 10.1007/s10654-022-00890-5 36260190 10.1007/s10654-022-00890-5 PMC9581448

[39] Ranasinghe P, Mathangasinghe Y, Jayawardena R, Hills AP, Misra A (2017) Prevalence and trends of metabolic syndrome among adults in the asia-pacific region: a systematic review. BMC Public Health 17:101. 10.1186/s12889-017-4041-1 28109251 10.1186/s12889-017-4041-1 PMC5251315

[40] Reisinger C, Nkeh-Chungag BN, Fredriksen PM, Goswami N (2021) The prevalence of pediatric metabolic syndrome-a critical look on the discrepancies between definitions and its clinical importance. Int J Obes (Lond) 45:12–24. 10.1038/s41366-020-00713-1 33208861 10.1038/s41366-020-00713-1 PMC7752760

[41] Rodríguez LA, Madsen KA, Cotterman C, Lustig RH (2016) Added sugar intake and metabolic syndrome in US adolescents: cross sectional analysis of the National Health and Nutrition Examination Survey 2005–2012. Public Health Nutr 19:2424–2434. 10.1017/S1368980016000057 26932353 10.1017/S1368980016000057 PMC10270843

[42] Safiri S, Karamzad N, Kaufman JS, Bell AW, Nejadghaderi SA, Sullman MJM, Moradi-Lakeh M, Collins G, Kolahi AA (2022) Prevalence, deaths and disability-adjusted-life-years (DALYs) due to type 2 diabetes and its attributable risk factors in 204 countries and territories, 1990–2019: results from the Global Burden of Disease Study 2019. Front Endocrinol (Lausanne) 13:838027. 10.3389/fendo.2022.838027 35282442 10.3389/fendo.2022.838027 PMC8915203

[43] Saklayen MG (2018) The global epidemic of the metabolic syndrome. Curr Hypertens Rep 20:12. 10.1007/s11906-018-0812-z 29480368 10.1007/s11906-018-0812-z PMC5866840

[44] Tropeano A, Corica D, Li Pomi A, Pepe G, Morabito LA, Curatola SL, Casto C, Aversa T, Wasniewska M (2021) The metabolic syndrome in pediatrics: do we have a reliable definition? A systematic review. Eur J Endocrinol 185:265–278. 10.1530/EJE-21-0238 34061767 10.1530/EJE-21-0238

[45] United Nations Educational Scientific and Cultural Organization (UNESCO) (2012) International Standard Classification of Education, ISCED 2011. Canada: UNESCO Institute for Statistics

[46] Wilkinson M, Dumontier M, Aalbersberg I et al (2016) The FAIR Guiding Principles for scientific data management and stewardship. Sci Data 3:160018. 10.1038/sdata.2016.18 26978244 10.1038/sdata.2016.18 PMC4792175

[47] Xu T, Liu J, Liu J, Zhu G, Han S (2017) Relation between metabolic syndrome and body compositions among Chinese adolescents and adults from a large-scale population survey. BMC Public Health 17:337. 10.1186/s12889-017-4238-3 28427375 10.1186/s12889-017-4238-3 PMC5397692

[48] Zhao Y, Yu Y, Li H, Li M, Zhang D, Guo D, Yu X, Lu C, Wang H (2019) The association between metabolic syndrome and biochemical markers in Beijing adolescents. Int J Environ Res Public Health 16:4557. 10.3390/ijerph16224557 31752150 10.3390/ijerph16224557 PMC6887991

